# Automated Stopped-Flow Fluorimetric Sensor for Biologically Active Adamantane Derivatives Based on Zone Fluidics

**DOI:** 10.3390/molecules24213975

**Published:** 2019-11-03

**Authors:** Paraskevas D. Tzanavaras, Sofia Papadimitriou, Constantinos K. Zacharis

**Affiliations:** 1 Laboratory of Analytical Chemistry, School of Chemistry, Faculty of Sciences, Aristotle University of Thessaloniki, Thessaloniki GR-54124, Greece; paristzanavaras@gmail.com; 2 Laboratory of Pharmaceutical Analysis, Department of Pharmaceutical Technology, School of Pharmacy, Aristotle University of Thessaloniki, Thessaloniki GR-54124, Greece; czacharis@pharm.auth.gr

**Keywords:** memantine, rimantadine, amantadine, zone fluidics, *o*-phthalaldehyde, derivatization, stopped-flow, quality control

## Abstract

A zone-fluidics (ZF) based automated fluorimetric sensor for the determination of pharmaceutically active adamantine derivatives, i.e., amantadine (AMA), memantine (MEM) and rimantadine (RIM) is reported. Discrete zones of the analytes and reagents (*o*-phthalaldehyde and *N*-acetylcysteine) mix and react under stopped-flow conditions to yield fluorescent *iso*-indole derivatives (λ_ex_/ λ_em_ = 340/455 nm). The proposed ZF sensor was developed and validated to prove suitable for quality control tests (assay and content uniformity) of commercially available formulations purchased from the Greek market (EU licensed) and from non-EU web-pharmacies at a sampling rate of 16 h^−1^. Interestingly, a formulation obtained through the internet and produced in a third—non-EU—country (AMA capsules, 100 mg per cap), was found to be out of specifications (mean assay of 85.3%); a validated HPLC method was also applied for confirmatory purposes.

## 1. Introduction

Adamantane analogues namely amantadine (AMA) and rimantadine (RIM) have been extensively used for the treatment of influenza A virus infection for more than three decades [1,2]. The mechanism of their action is based on the inhibition of influenza A virus reproduction by hindering the M2 protein ion channel and thus preventing the release of viral RNA into the cytoplasm of infected cells [3,4]. On the other hand, another structurally analogous compound, memantine (MEM), is a noncompetitive N-methyl-D-aspartate receptor antagonist is widely used for the treatment of Alzheimer’s and Parkinson’s diseases, dementia syndromes and also more recently for the possible treatment of glaucoma [5,6].

Quality control is an important step during and after the production process of pharmaceutical formulations to ensure the safety and therapeutic efficacy of the final products. Additionally, due to the evolution of WEB drug suppliers and the existence of fake products in the market, the QC of commercially available pharmaceuticals is even more critical. On this basis, automation of the analytical process offers significant advantages in terms of rapidity and effectiveness [7]. The concept of Zone Fluidics (ZF) [8] combines some interesting features that are more than welcome in pharmaceutical analysis: (i) automation of chemical reactions through zones, (ii) single line configurations, (iii) generation of waste at the level of microliters, (iv) no need for reconfiguration in order to apply different methods, (v) robustness and unattended long term operation, etc. 

A literature search (Scopus) revealed only a few automated flow methods reporting the determination of adamantine derivatives [9,10,11,12,13]. Most of them are based on potentiometric detection through suitable electrodes [9,10,11]. Although such approaches are interesting from an academic point of view, when it comes to real world QC industrial applications, the main disadvantage is the non-commercial availability of the electrodes. Other unattractive features include the fouling of the electrode surface in real sample analysis, the necessity for periodic activation/conditioning of the electrode [9], logarithmic regression equations, while only in the memantine potentiometric method proposed by Nashar et al., the authors presented validation results according to the international guidelines (i.e., robustness, ruggedness, etc.) [11]. On the other hand, in the chemiluminescence-based method reported by Alarfaj and El-Tohamy, the authors selected only the pH to validate the robustness of the SI procedure when, from the schematic effects of the other parameters, it is obvious that small variations in the flow rate, the aspiration volumes or the concentration of the reagents cause dramatic variations in the CL responses [12]. Finally, a more recent fluorimetric method proposed for the determination of amantadine is based on a competitive assay between the drug and the dye Thionine for the Cucurbit [8], uril cavity [13]. Although the authors claim adequate analytical features for application in both pharmaceuticals and urine samples, no proper validation experiments were presented to support the effectiveness of the procedure for QC purposes (i.e., robustness, determination range, residuals, etc.).

From an analytical chemistry point of view, the bottleneck for the development of simple methods for the direct quantitation of this class of compounds is the lack of chromophore or fluorophore moieties in their chemical structure. Derivatization through the primary amino group is therefore a viable solution in order to enhance their detectability. Among the various reagents that exist for the derivatization of primary amines [14], in this study, we selected *o*-phthalaldehyde (OPA) for the following reasons: (i) it reacts fast and under mild conditions, (ii) it is commercially available at low/reasonable cost, (iii) it is stable and simple to handle, (iv) it typically offers adequately sensitivity for most applications; on the other hand—compared to other reagents—OPA derivatives are less stable, but this is not a disadvantage when the reactions and detection are carried out on-line in a strictly reproducible analytical cycle [15].

The goals of the present study where, therefore, the following: (i) to examine the reaction of OPA with the three pharmaceutically active adamantine derivatives under flow conditions; (ii) to combine the advantages of OPA and the ZF automated technique; (iii) to develop and validate a simple and efficient platform for the analysis of pharmaceuticals from various sources in order to prove their safety and therapeutic efficacy.

## 2. Results and Discussion

### 2.1. Reactivity of MEM, AMA, RIM with o-Phthalaldehyde

Preliminary experiments confirmed that the analytes can react with OPA through their primary amino group under flow conditions, in the presence of a suitable nucleophilic agent (NAC) (Figure S2 in the supplementary material). However, it was interesting to notice that despite the structure similarities of the analytes, RIM showed a considerably higher reactivity in both terms of reaction speed and sensitivity, due to the fact that the primary amino group is attached to a ternary carbon atom. The order of reactivity (RIM >> AMA ≅ MEM) is in accordance with previous reports on the behavior of primary aliphatic amines and is affected mainly by steric hindrance and polar phenomena [16,17]. 

In order to improve the sensitivity of the analytical procedure, preliminary experiments were also focused on a stopped-flow step prior to FL detection. The advantage of the stopped-flow technique is the minimization of the radial dispersion effect that results in broad peaks and competes to the sensitivity gain due to the increased reaction time. Stopped-flow can be either applied in the flow cell or in a suitable coil before detection. The former approach is advantageous on the basis of monitoring the reaction in real time. However, our experiments proved that the OPA derivatives were not stable under the flow-cell conditions (e.g., light and temperature) [18]. For this reason, the stopped-flow step was carried out in a reaction coil (RC, 60 cm/0.5 mm i.d.) prior to entering the detector.

### 2.2. Development of the ZF Sensor

The development of the ZF sensor involved experimental studies of the various chemical and instrumental parameters that are expected to affect the performance of the procedure. The investigated parameters, the studied range and the finally selected values are tabulated in Table 1. The selection criteria were a compromise among sensitivity, sampling rate and reagent consumption. The starting values of the variables during experiments were: *c*(OPA) = 1.0 mmol L^−1^, *c*(NAC) = 1.0 mmol L^−1^ in pH = 10.0 (0.1 mol L^−1^ carbonate buffer), *γ*(ΑTD) = *γ*(ΜΕΜ) = 100 mg L^−1^, *γ*(RIM) = 10 mg L^−1^, *Q*_V_(C) = 0.6 mL min^−1^, *V*(AMA) = *V*(MEM) = *V*(RIM) = *V*(OPA) = *V*(NAC) = 50 μL. The order of zones mixing had no practical effect on the analysis, and the sequence described in Section 2.3 was adopted (NAC/Buffer–OPA–Sample).

The effect of the stopped flow time was examined in the range of 0–180 s. The signals increased non-linearly in the studied range, with the sensitivity increasing 2-fold in the first 60 s for all analytes. The latter value was selected making a compromise between sensitivity and sampling rate.

**Figure 1:**
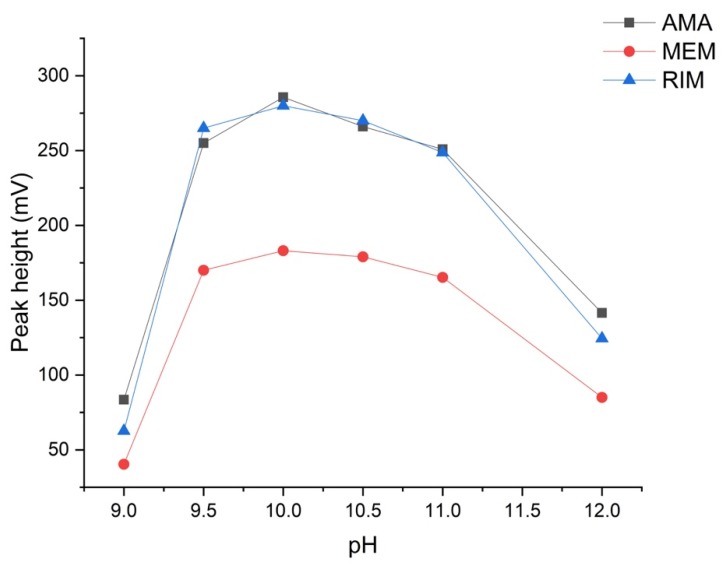
Effect of the pH on the derivatization reaction; for experimental details see Section 2.2. AMA = amantadine, RIM = rimantadine, MEM = memantine.

The pH proved to be a critical variable for all compounds. Its effect was evaluated in the range of 9.0–12.0, using 100 mmol L^−1^ carbonate buffer. As can be seen in the experimental results in Figure 1, a clear maximum at a pH value of 10 was observed for all analytes. The type of buffer proved to have no effect (carbonate vs borate vs Britton–Robinson).

The effects of the volume and amount concentration of the OPA and NAC reagents were investigated within the ranges included in Table 1. The criteria for selecting the final values were sensitivity and consumption. In all cases non-linear relationships were observed between the fluorescence intensities and the examined variables. The values of 75 μL (NAC solution, 2.5 mmol L^−1^) and 50 μL (OPA solution, 5 mmol L^−1^) provided adequate sensitivity and excess of reagents for the selected applications. 

The effect of the sample injection volume is always an important parameter in flow-based sensors since it directly affects the dispersion of the analytes within the flow lines. The sensitivity increased between 150% and 200% for all analytes within 50–150 μL sample injection volume. Finally, the volume of 100 μL was selected as a compromise between sensitivity, consumption and sampling rate.

### 2.3. Validation of the ZF Sensor

The proposed sensor has been validated for determination range, limits of detection (LOD) and quantification (LOQ), precision, selectivity, accuracy and robustness.

The determination range was evaluated within 50% to 150% of the 100% level for the three analytes, i.e., 50–150 mg L^−1^ for AMA and MEM and 5–15 mg L^−1^ for RIM. The selected range covers the specifications for both assay and content uniformity tests. Six calibration levels were used (*n* = 6). The respective regression equations were

*F* = 72.9 (±5.8) + 4.84 (±0.05) × *γ*(AMA), *r*^2^ = 0.9996

*F* = 100.8 (±3.0) + 2.56 (±0.03) × *γ*(MEM), *r*^2^ = 0.9996

*F* = 17.0 (±6.1) + 42.28 (±0.95) × *γ*(RIM), *r*^2^ = 0.9990

where *F* is the fluorescence intensity as measured by the detector and *γ* the mass concentration of the analytes in mg L^−1^. Linearity was further validated using the back-calculated concentrations (residuals). The percent residuals were distributed randomly around the “zero axis” and ranged < ± 3% (−1.5% to +0.9% for AMA, −0.2% to +1.1% for MEM and −1.6% to +1.7% for RIM).

The LODs and LOQs were estimated based on the following equations

LOD = 3.3 × SD_b_/*s* and LOQ = 10 × SD_b_/*s*

where SD_b_ is the standard deviation of the intercept and s is the slope of the respective regression lines. The calculated LOD/LOQ for the three analytes were 4/12 mg L^−1^ for AMA, 3.9/11.2 mg L^−1^ for MEM and 0.5/1.5 mg L^−1^ for RIM respectively. 

The within-day precision was validated at the 100% level for the three active pharmaceutical ingredients, i.e., 100 mg L^−1^ for AMA and MEM and 10 mg L^−1^ for RIM. The RSD values were better than 1.9% for 8 consecutive injections. The day-to-day precision was validated by comparing the slopes of six regression lines for each analyte within a time period of 10 days. The RSD values of the slopes were better than 6.5% in all cases.

The selectivity and accuracy of the proposed sensor were validated using the “placebo approach” by preparing synthetic samples at three concentration levels of 50%, 100% and 150% for each compound. The synthetic samples also contained the placebo mixture (all excipients except for the active ingredients) at 1000 mg L^−1^ for AMA and MEM and 100 mg L^−1^ for RIM (10-fold excess over the 100% level). The acceptable recoveries should be in the range of 95% to 105%. The experimental results included in Table 2 confirmed the accuracy and selectivity of the procedure.

The robustness of the proposed method was evaluated by small deliberate variations (±5%) on critical instrumental and chemical parameters such as the stopped flow time (57–63 s), the sample volume (95–105 μL), the reaction pH (9.5–10.5), the amount concentration of OPA (4.7–5.3 mmol L^−1^) and the amount concentration of NAC (2.3–2.7 mmol L^−1^). With the exception of the pH all examined parameters resulted in variations in the range of 95% to 105% for all analytes. On the other hand, the strict regulation of the pH proved to be the most critical prerequisite since the variations ranged between 91.5% to 110.8%.

### 2.4. Applications of the ZF Sensor

**Figure 2:**
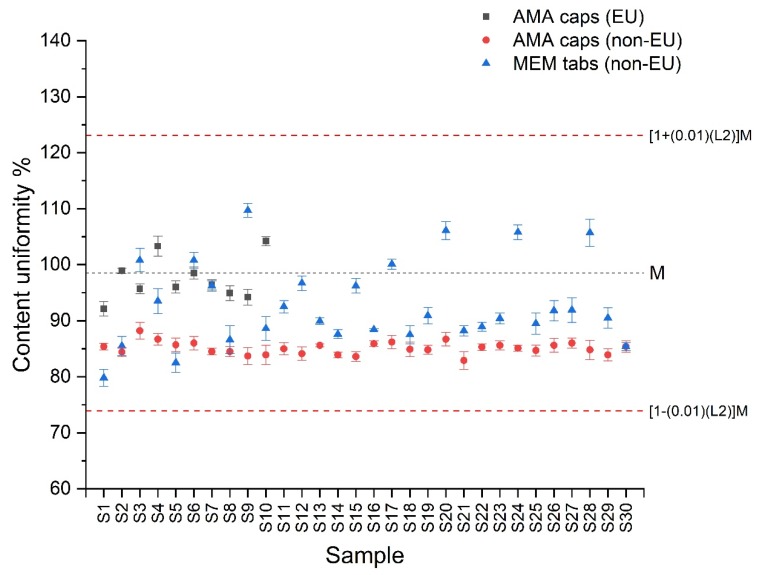
Graphical depiction of the content uniformity results of the selected EU and non-EU formulations using the proposed ZF method; LSL = lower specification limit, USL = upper specification limit.

The developed ZF fluorimetric sensor has been applied to the quality control (QC) of commercially available AMA and MEM containing pharmaceuticals (there were no commercially available RIM formulations in the Greek market). Two of the formulations, i.e., MEM oral drops (10 mg mL^−1^) and AMA caps (100 mg per cap) were EU-licensed obtained from the Greek market, while two additional formulations, i.e., MEM tabs (5 mg per tab) and AMA(2) caps (100 mg per tab) were obtained from web-pharmacies and are produced in third countries (non-EU licensed). MEM oral drops were analyzed in terms of assay of the active ingredient, while capsules and tablets were also analyzed for content uniformity. The experimental results are presented in Table 3 and Table 4. The Tables also include the specification limits of the pharmacopoeias for each formulation and the results from a corroborative, in-house validated HPLC method based on reversed phase separation and post-column derivatization (see Section 3.5 for details). The comparison of the obtained results (*t*-test) indicated that there is no statistical difference between the proposed ZF and HPLC methods at confidence interval 95% (*t*_exp_ < *t*_crit (95%, *n* = 4)_). The content uniformity results are also presented graphically, including the specification limits according to USP monograph (Figure 2).

As can be seen in the experimental results of Table 3, both EU-licensed formulations and the non-EU MEM tablets were within specifications regarding to the assay test. On the other hand, the non-EU licensed AMA capsules were out of specifications in the assay test with an average content of 85.3 (±2.1) mg AMA per capsule that is less than the 90% to 110% limits set by international pharmacopoeias. These results were found to be in accordance with the corroborative HPLC method. As can be seen in Table 4, only the EU-licensed AMA capsule formulation passed the content uniformity test based on the USP acceptance value (AV). Non-EU produced AMA and MEM formulations failed to pass the content uniformity test using both 10 and 30 units for analysis. 

## 3. Materials and Methods 

### 3.1. Instrumentation

**Figure 3:**
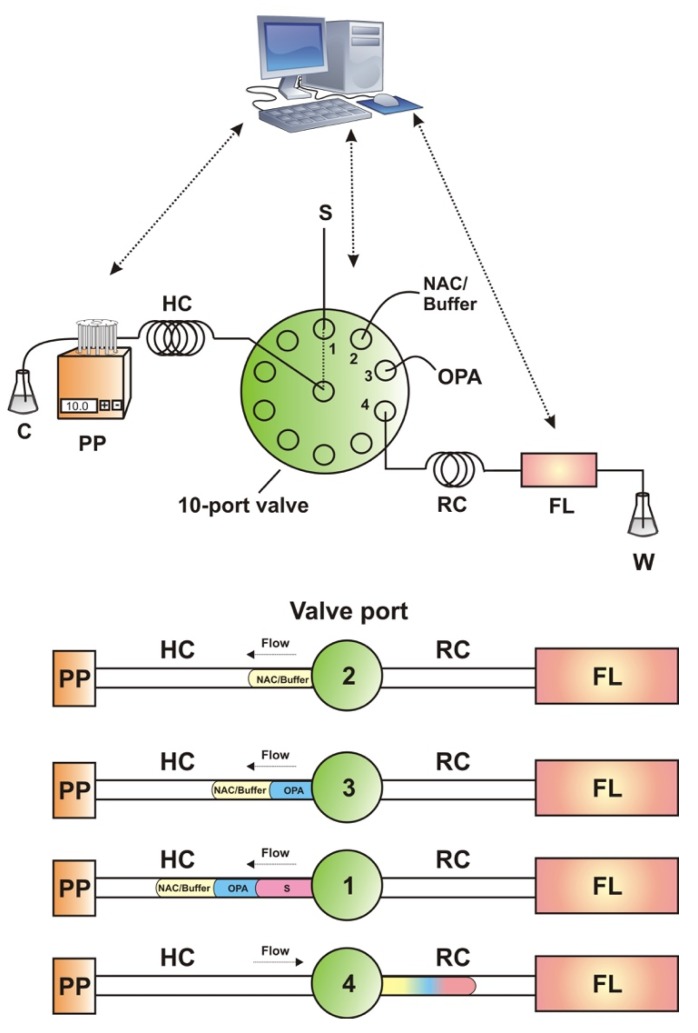
Graphical depiction of the Zone Fluidics manifold and the analytical sequence steps for the determination of the adamantane derivatives; PP = peristaltic pump, HC = holding coil, S = sample, FL = fluorimetric detector, RC = reaction coil, C = carrier (water), W = waste, OPA = *o*-phthalaldehyde, NAC = *N*-acetylcysteine.

The home-made ZF setup (Figure 3) consisted of the following parts: a Minipuls3 peristaltic pump (Gilson), a micro-electrically actuated 10-port valve (Valco), a RF-551 flow-through spectrofluorimetric detector (Shimadzu); PTFE tubing was used for the connections of the flow configuration (0.5 or 0.7 mm i.d.), and Tygon tubing was used in the peristaltic pump. Control of the ZF system was performed through a LabVIEW (National Instruments) based program also developed in house. 

The HPLC-PCD setup comprised the following parts (Figure S1 in supplementary material): a AS3000 autosampler (Thermo Scientific), a LC-9A binary pump (Shimadzu, Japan), a RF-551 spectrofluorimetric detector (λ_ex_/λ_em_ = 340/455 nm) (Shimadzu, Japan), an Elite^TM^ vacuum degasser (Alltech, U.S.), a 150 × 4.6 mm i.d. reversed-phase column (Prevail, Alltech). A peristaltic pump was employed for the propulsion of the PCD reagents (Gilson Minipuls3). PTFE tubing (0.5 mm i.d.) was used for the construction of the reaction coil (RC) and all necessary PCD connections.

Data acquisition including peak highs (ZF method) and areas (HPLC-PCD method) was carried out through the Clarity^®^ software (version 4.0.3, DataApex, Czech Republic).

### 3.2. Reagents and Solutions

Memantine (MEM), Amantadine (AMA) and Rimantadine (RIM) were purchased by Sigma; *o*-phthalaldehyde (OPA, Fluka), *N*-acetylcysteine (NAC, Merck), Na_2_CO_3_ (Merck), NaOH (Merck) and HCl (Sigma) were all of analytical grade. Doubly de-ionized water was produced by a Milli-Q system (Millipore). 

The stock solutions of the analytes were prepared at the 1000 mg L^−1^ level in water. They were kept refrigerated and were found to be stable for at least one month. Working solutions were prepared on a daily basis by appropriate dilutions in water. The 100% level was 100 mg L^−1^ for MEM and AMA and 10 mg L^−1^ for RIM. 

The derivatizing reagent (OPA) was prepared at an amount concentration of 10 mmol L^−1^ by firstly dissolving in 0.5 mL methanol and subsequently adding 9.5 mL water [19]. This solution was stable for two weeks at 4 ^o^C in an aluminum foil-wrapped container. NAC was prepared in water (10 mmol L^−1^) and diluted at the desired working concentrations in 100 mmol L^−1^ carbonate buffer (pH = 10). 

The placebo mixture used in accuracy and selectivity studies consisted of representative pharmaceutical grade excipients and was prepared at a nominal concentration of 10 mg mL^−1^ according to previously published protocols [20].

### 3.3. ZF Procedure

A graphical depiction of the ZF sequence can be found in Figure 3. In brief, NAC/Buffer (75 μL, 2.5 mmol L^−1^, pH = 10), OPA (50 μL, 5 mmol L^−1^) and sample (100 μL) were sequentially aspirated in the holding coil (HC). The reaction mixture was propelled for 30 s towards the reaction coil (RC) at a flow rate of 0.6 mL min^−1^, and the reaction was allowed to develop for 60 s under stopped-flow conditions. Detection was carried out downstream at λ_ex_/λ_em_ = 340/455 nm. More detailed description of the ZF steps is tabulated in Table S1 (supplementary material). The sampling throughput was 16 h^−1^. 

### 3.4. Preparation of Samples

Pharmaceutical samples included both EU-licensed products—purchased from local pharmacies—and non-EU-licensed formulations—purchased from web-pharmacies. Three lots of MEM oral drops (10 mg mL^−1^) were analyzed directly, after 10-fold dilution in water. AMA capsules (100 mg per cap) and MEM tablets (5 mg per tab) (*n* = 10) were ultrasonically dissolved in water followed by filtration through 0.45 μm syringe filters and dilution with water to meet the 100% level concentration. For content uniformity tests, ten capsules or tablets from each formulation were treated individually (*n* = 10) as indicated by the U.S. pharmacopoeia [21].

### 3.5. HPLC-PCD Corroborative Method

Fifty microliters of samples or standards were injected in HPLC column and separated at ambient temperature at a flow rate of 0.5 mL min^−1^. The HPLC mobile phase was CH_3_OH/phosphate buffer (25 mmol L^−1^, pH = 2.0) at a volume ratio of 50:50. Prior to use, it was filtered under vacuum through 0.22 μm membrane filters (Whatman^®^). The post-column-derivatization reagents were OPA at an amount concentration of 20 mmol L^−1^ and a mixture of NAC (5 mmol L^−1^)/100 mmol L^−1^ borate buffer (pH = 11.0). The PCD reagents were pre-mixed on-line (0.2 mL min^−1^ each) through a binary inlet static mixer (BISM, ASI-Analytical Scientific Instruments) with internal volume of 250 μL. The derivatization was allowed to proceed on passage through a 100 cm long knitted reaction coil. The derivatives of Amantadine, (*t*_R_ = 6.6 min), Rimantadine (*t*_R_ = 13.5 min), Memantine (*t*_R_ = 15.8 min) were detected fluorimetrically at *λ*_ex_/*λ*_em_ = 340/455 nm (see typical chromatogram in Figure S3 in the supplementary material).

## 4. Conclusions

The developed automated fluorimetric sensor for the analysis of pharmaceutically active adamantane derivatives offers some interesting features:

1. This is the first flow-based method for this class of active pharmaceutical ingredients;
2. The sensor utilizes commercially available reagents, is simple and straightforward;
3. The concept of zone fluidics minimizes the consumption of the samples and reagents compared to continuous flow methods, at a sampling rate of 16 h^−1^;
4. Validation experiments confirmed the suitability of the method for the QC of commercially available formulations;
5. A non-EU licensed formulation was found to be out of the specifications set by international pharmacopoeias;

## Figures and Tables

**Table 1:** Study of instrumental and chemical variables.

Variable	Studied Range	Selected Value
*Instrumental*		
*t* (SF)/s	0–180	60
*V*(S)/μL	50–100	100
*V*(R)/μL	25–100	75
*V*(OPA)/μL	25–100	50
*Chemical*		
pH	9.0–12.0	10.0
*c*(NAC)/mmol L^−1^	0.5–5.0	2.5
*c*(ΟΡΑ)/mmol L^−1^	0.5–10.0	5.0

**Table 2:** Accuracy and selectivity of the proposed ZF method.

Sample	Analyte Concentration (mg L^−1^)	Placebo Concentration (mg L^−1^)	Recovery (%)	
			AMA	MEM
**S1**	50	1000	103.6	97.2
**S2**	100	1000	101.0	99.7
**S3**	150	1000	102.9	101.7
			**RIM**	
**S1**	5	100	103.6	
**S2**	10	100	97.1	
**S3**	15	100	101.5	

**Table 3:** Assay of AMA- and MEM-containing pharmaceutical formulations.

Formulation/Sample	Label Content	Specifications	Assay (%)	HPLC (%)
AMA caps (EU)	100 mg/cap	95% to 105%	97.4 (±1.5 ^a^)	96.1 (±2.2)
AMA caps (non-EU)	100 mg/cap	95% to 105%	85.3 (±1.2)	86.9 (±1.8)
MEM tabs (non-EU)	5 mg/tab	95% to 105%	92.4 (±1.7)	92.2 (±1.9)
MEM oral drops (EU)				
Lot1	10 mg mL^−1^	95% to 105%	99.3 (±0.8)	100.5 (±1.7)
Lot2	10 mg mL^−1^	95% to 105%	99.5 (±1.2)	101.2 (±2.6)
Lot3	10 mg mL^−1^	95% to 105%	98.5 (±1.1)	99.8 (±2.3)

^a^ Standard deviation.

**Table 4:** Content/Dosage uniformity test of pharmaceutical preparations.

Sample	Content Uniformity (%)		
	AMA Caps (EU) 100 mg/Cap	AMA Caps (Non-EU) 100 mg/Cap	MEM Tabs (Non-EU) 5 mg/Tab
**S1**	92.1 (±1.3 ^a^)	85.4 (±0.6)	79.8 (±1.5)
**S2**	98.9 (±0.5)	84.4 (±0.8)	85.5 (±1.7)
**S3**	95.7 (±0.9)	88.2 (±1.5)	100.8 (±2.1)
**S4**	103.3 (±1.8)	86.7 (±1.0)	93.5 (±2.2)
**S5**	96.0 (±1.1)	85.7 (±1.2)	82.5 (±1.7)
**S6**	98.5 (±1.1)	86.0 (±1.2)	100.8 (±1.4)
**S7**	96.4 (±0.9)	84.5 (±0.6)	96.2 (±0.9)
**S8**	94.9 (±1.3)	84.5 (±0.9)	86.6 (±2.5)
**S9**	94.2 (±1.4)	83.7 (±1.5)	109.7 (±1.2)
**S10**	104.2 (±0.8)	83.9 (±1.7)	88.6 (±2.1)
**S11**	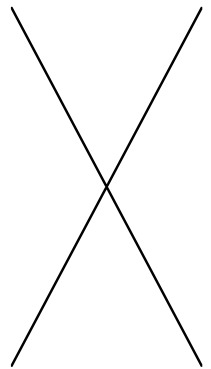	85.0 (±1.1)	92.5 (±1.1)
**S12**		84.1 (±1.2)	96.7 (±1.3)
**S13**		85.6 (±0.3)	89.9 (±0.6)
**S14**		83.9 (±0.5)	87.6 (±0.8)
**S15**		83.6 (±0.9)	96.2 (±1.3)
**S16**		85.9 (±0.5)	88.4 (±0.2)
**S17**		86.2 (±1.2)	100.1 (±0.9)
**S18**		84.9 (±1.3)	87.5 (±1.6)
**S19**		84.8 (±0.8)	90.9 (±1.5)
**S20**		86.7 (±1.2)	106.1 (±1.6)
**S21**		82.9 (±1.6)	88.2 (±0.9)
**S22**		85.3 (±0.6)	88.9 (±0.8)
**S23**		85.6 (±0.8)	90.4 (±1.0)
**S24**		85.1 (±0.5)	105.8 (±1.3)
**S25**		84.7 (±1.0)	89.5 (±1.9)
**S26**		85.6 (±1.2)	91.8 (±1.8)
**S27**		86.0 (±0.9)	91.9 (±2.2)
**S28**		84.8 (±1.7)	105.7 (±2.4)
**S29**		83.9 (±1.1)	90.5 (±1.8)
**S30**		85.4 (±1.0)	85.4 (±0.7)
**X**	97.4	85.1	92.9
**M**	98.5	98.5	98.5
**s**	3.9	1.1	7.4
**AV**	10.5	15.6	20.5
**L1**	15	15	15
Result	Pass (AV<L1)	Fail (AV>L1)	Fail (AV>L1)

^a^ Standard deviation.

## Supplementary Materials

The following are available online at https://www.mdpi.com/1420-3049/24/21/3975/s1, Figure S1: Schematic diagram of the HPLC-PCD setup; Figure S2: Reaction between OPA and the studied adamantine derivatives using N-acetylcysteine (NAC) as nucleophilic reagent, Figure S3: Representative chromatogram from standard mixture of the adamantane derivatives by the corroborative HPLC-PCD method, Table S1: ZF sequence for the automated determination of adamantane derivatives.
