# Supplementary material for: Automated Stopped-Flow Fluorimetric Sensor for Biologically Active Adamantane Derivatives Based on Zone Fluidics
Source: Molecules. 2019 Nov 3;24(21):3975. doi: 10.3390/molecules24213975 (PMC6864522; doi:10.3390/molecules24213975)
Supplement: Supplementary file 1 [file molecules-24-03975-s001.pdf]

Automated Stopped-Flow Fluorimetric Sensor for Biologically Active Adamantane Derivatives Based on Zone Fluidics

Paraskevas D. Tzanavaras*[1], Sofia Papadimitriou[1] and Constantinos K. Zacharis[2]

[1] Laboratory of Analytical Chemistry, School of Chemistry, Faculty of Sciences,
Aristotle University of Thessaloniki, GR-54124, Greece
[2] Laboratory of Pharmaceutical Analysis, Department of Pharmaceutical Technology, School of Pharmacy,
Aristotle University of Thessaloniki, GR-54124, Greece

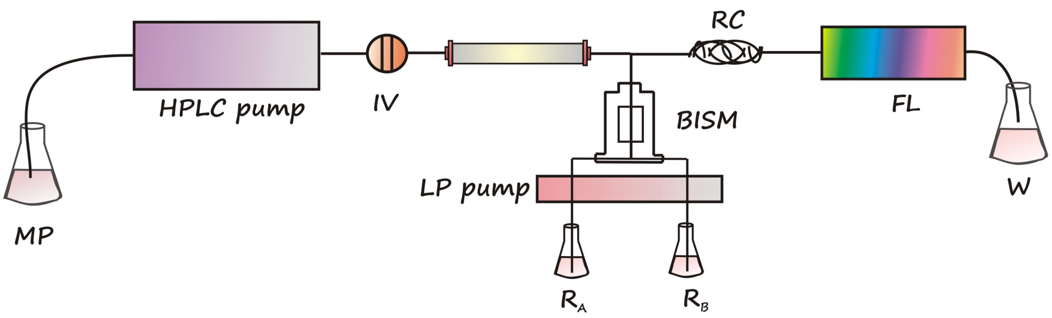

Figure S1. Schematic diagram of the HPLC-PCD setup: MP = mobile phase [50:50 v/v $CH_3OH:NaH_2PO_4$ (25 mmol L^{-1}, pH = 3.0)]; IV = autosampler (V = 50 μL); C = HPLC column (Prevail C18, 100 × 4.6 mm i.d.); BISM = Binary Inlet Static Mixer; LP pump = low-pressure pump; RC = knotted reaction coil (100 cm / 0.5 mm i.d.); PP = peristaltic pump; R_A = OPA (c = 20 mmol L^{-1}, Q_V = 0.2 mL min^{-1}); R_B = NAC (5 mmol L^{-1}) / borate buffer (100 mmol L^{-1}, pH = 11.0, Q_V = 0.2 mL min^{-1}); FL = Fluorescence detector ($\lambda_{ex}/\lambda_{em}$ = 340 / 455 nm).

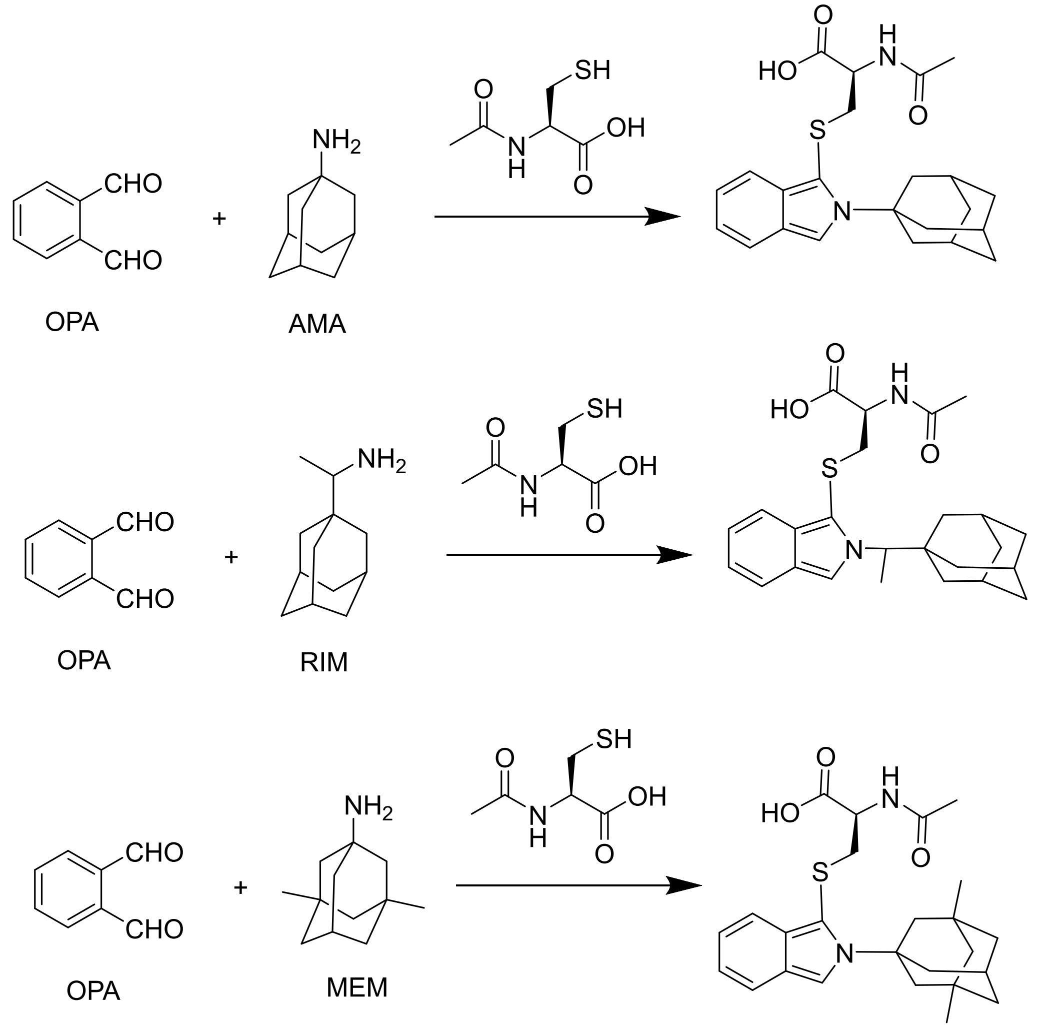

Figure S2. Reaction between OPA and the studied adamantine derivatives using N-acetylcysteine (NAC) as nucleophilic reagent; AMA = amantadine, RIM = rimantadine, MEM = memantine.

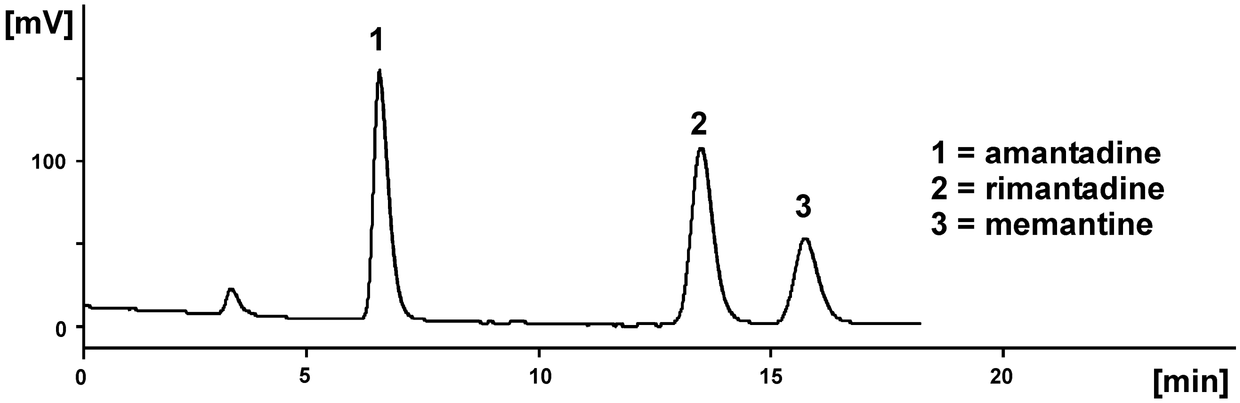

Figure S3. Represenatative chromatogram from standard mixture of the adamantane derivatives by the corroborative HPLC-PCD method; for experimental details please see sections 2.1 and 2.5.

Table S1. ZF sequence for the automated determination of adamantane derivatives.

a/a	Time (*s*)	Valve position	Pump action	Flow rate (mL min^{-1})	Volume (μL)	Action description
1	1	2	Off	–	–	Selection of NAC/Buffer port
2	7.5	2	Aspirate	0.6	75	Aspiration of NAC/Buffer in the HC
3	1	3	Off	–	–	Selection of OPA port
4	5	3	Aspirate	0.6	50	Aspiration of OPA in the HC
5	1	1	Off	–	–	Selection of sample port
6	10	1	Aspirate	0.6	100	Aspiration of sample in the HC
7	1	4	Off	–	–	Selection of FL detector port
8	30	4	Deliver	0.6	300	Deliver of the reaction mixture to the RC
9	60	4	Off	–	–	Stop-flow to proceed the reaction
10	120	4	Deliver	0.6	600	Deliver of the reaction mixture to the FL detector
